# Impact of Low Social Preference on the Development of Depressive and Aggressive Symptoms: Buffering by Children’s Prosocial Behavior

**DOI:** 10.1007/s10802-017-0382-6

**Published:** 2017-12-19

**Authors:** Jin He, Hans M. Koot, J. Marieke Buil, Pol A. C. van Lier

**Affiliations:** 1 Section Clincial Developmental Psychology, Vrije Universiteit Amsterdam, Van der Boechorststraat 1, 1081 BT Amsterdam, The Netherlands; 2 Department of Psychology, Erasmus University Rotterdam, Burgemeester Oudlaan 50, 3062 PA Rotterdam, The Netherlands; 3 EMGO Institute for Health and Care Research, van der Boechorststraat 7, 1081 BT Amsterdam, The Netherlands

**Keywords:** Social preference, Prosocial behavior, Depression, Aggression, Childhood

## Abstract

Holding a low social position among peers has been widely demonstrated to be associated with the development of depressive and aggressive symptoms in children. However, little is known about potential protective factors in this association. The present study examined whether increases in children’s prosocial behavior can buffer the association between their low social preference among peers and the development of depressive and aggressive symptoms in the first few school years. We followed 324 children over 1.5 years with three assessments across kindergarten and first grade elementary school. Children rated the (dis)likability of each of their classroom peers and teachers rated each child’s prosocial behavior, depressive and aggressive symptoms. Results showed that low social preference at the start of kindergarten predicted persistent low social preference at the start of first grade in elementary school, which in turn predicted increases in both depressive and aggressive symptoms at the end of first grade. However, the indirect pathways were moderated by change in prosocial behavior. Specifically, for children whose prosocial behavior increased during kindergarten, low social preference in first grade elementary school no longer predicted increases in depressive and aggressive symptoms. In contrast, for children whose prosocial behavior did not increase, their low social preference in first grade elementary school continued to predict increases in both depressive and aggressive symptoms. These results suggest that improving prosocial behavior in children with low social preference as early as kindergarten may reduce subsequent risk of developing depressive and aggressive symptom.

Peer relationships become increasingly important for children’s psychological adjustment as they enter into kindergarten (Keiley et al. 2000; Ladd et al. 2006; Parker et al. 2006). A number of longitudinal studies demonstrated that children with peer relationship difficulties in school are at risk of developing symptoms of both depression and aggression (Brendgen et al. 2002; Cillessen and Mayeux 2004; Sturaro et al. 2011). Moreover, early experiences of peer difficulties, even during kindergarten, are likely to repeat and become persistent through childhood and even older ages (Jiang and Cillessen 2005). In particular, children with *chronic* peer difficulties incur the risk of developing symptoms of depression and aggression (Burks et al. 1995; DeRosier and Janis 1994; Ladd and Troop-Gordon 2003). Although we know that chronic peer difficulties in childhood are linked to maladjustment, our knowledge on factors that may protect children from developing persistent peer difficulties and associated negative outcomes is scarce (Cicchetti and Natsuaki 2014). One of the behavioral strategies that may protect children against experiencing prolonged peer relationship difficulties and developing psychopathology could be to act more prosocially towards their peers, such as comforting others and being kind (Williams 2009). To test this idea, the present study followed 324 children from kindergarten through first grade elementary school, and explored whether children who increase their prosocial behavior in kindergarten are protected against the potential impact of low social preference on the development of depressive and aggressive symptoms.

Children who score low on social preference among peers may feel dissatisfied and lonely (Boivin et al. 1995; Fontaine et al. 2009). Being less preferred by peers may also reduce their opportunities to learn social norms and practice social skills in peer context (Rubin et al. 2006), which again, may aggravate their risk of being less liked. Unsurprisingly, with these associated disadvantages, low social preference was found to increase children’s risk of developing psychopathological symptoms (Haselager et al. 2002; Hoglund and Chisholm 2014; Ladd 2006; Van Lier and Koot 2010). Nonetheless, studies have suggested that some children may escape from the risk associated with low social preference. For example, it has been demonstrated that maintaining supportive relationship with parents (Bilsky et al. 2013) or other people in the school environment, especially those who are important for the child such as teachers (Spilt et al. 2014) and close friends (Bollmer et al. 2005; Laursen et al. 2007), weakens the link between low social preference and children’s psychopathologic symptoms. This indicates that positive interpersonal relationships can protect children with low peer social preference from developing maladaptive outcomes.

The impact of low social preference – and its possible repair – may be especially visible in those who are actively rejected by their peers. In line with the empirical studies mentioned above, ostracism theory suggests that behaviors promoting interpersonal connections can prevent rejected individuals from developing psychopathologic symptoms (Williams 2007, 2009). According to this theory, social rejection causes psychopathologic symptoms because it threatens the individual’s fundamental needs, including the need to belong, to maintain self-esteem, to perceive control over one’s social environment, and the need for a meaningful existence (Williams 2009). The threats to fundamental needs elicit a sequence of defensive behaviors in affected individuals to remove these threats. One of the ways to remove the threats is to rebuild the interrupted interpersonal connections by an increase in prosocial behavior (Williams 2009), such as being kind towards others. Ostracism theory suggests that differences exist in individuals’ attempts or abilities to adaptively increase prosocial behavior, which consequently affects the developmental outcomes (Williams 2009). If the prosocial attempt is successfully employed, individuals can make themselves more interpersonally attractive, thus preventing the initial rejection from becoming prolonged. Conversely, if the individual fails to display adaptive prosocial behavior, the rejection becomes persistent, thereby increasing the risk of developing psychopathologic symptoms. In short, an adaptive increase in prosocial behavior may be a key factor in preventing rejection from becoming prolonged and thereby prevent the development of psychopathologic symptoms (Williams 2009).

Despite the empirical evidences described above and the theoretical model proposed by Williams, to our knowledge, studies examining the potential buffering effect of increase in prosocial behavior in kindergarten and early elementary school are scarce. Compared to preschool, when children begin formal education, the time they spend with peers (Lam et al. 2014) and the size of peer groups both dramatically increase, while the availability of adults for guidance or supervision of peer social interactions is reduced (Rubin et al. 2006). Most of the children who enter the formal school setting have not experienced these challenges before (Ladd et al. 2006). Meanwhile, children’s poor preference among peers is found to be stable throughout the elementary school period (Ladd 2006). Importantly, peer difficulties, such as low social preference, have been shown to affect children’s symptoms of depression and aggression from kindergarten onward (Gooren et al. 2011). This effect may even last through adolescence (Will et al. 2016) and adulthood (Modin et al. 2010), especially when peer rejection becomes persistent. Thus, it is critical to raise knowledge about the potential factors that may reduce the impact of chronic peer difficulties.

Therefore, the goal of the present study is to explore the potential protective effect of increase in prosocial behavior in the link between low social preference and the development of depressive and aggressive symptoms. We had the following hypotheses: (1) increase in prosocial behavior during kindergarten would improve children’s social preference from kindergarten to the beginning of the first grade; and (2) the increase in prosocial behavior during kindergarten would mitigate the link between low social preference in early first grade and the development of depressive symptoms and aggressive behavior from kindergarten to the end of first grade in elementary school. To check whether our hypothesized effects apply equally to boys and girls, sex differences in the tested associations were also examined.

## Method

### Participants

Data were collected from 18 schools in the north and east of the Netherlands as part of a longitudinal project on children’s social and emotional development. Schools were recruited through the Dutch Municipal Health Service (MHS), and the first 18 schools willing to join were included in the longitudinal project. This study included all children who were in kindergarten at the beginning of the project (*N* = 324, 54% boys; *M*_age_ = 5.10, *SD* = 0.37 at baseline). The majority (95.2%) of children were Dutch/Caucasian, 3.1% were Turkish, 0.3% were Surinamese, and 1.4% belonged to other ethnic groups.

All children were followed from kindergarten to the end of first grade elementary school. The first assessment was conducted in the fall of kindergarten (T1). The second and third assessments were conducted in the fall (T2) and spring (T3) of the first grade in elementary school. Before each assessment, parents were informed about the procedure and measurements, and were given the opportunity to decline their children’s participation in the study. Children were also informed in class and had the opportunity to decline their participation at any time during the study. Almost all invited children (99.9%) participated. After each assessment, a small gift was given to the children as a token of appreciation for their participation. The study proceedings were approved by the Medical Ethical Review Board of the VU Medical Centre.

Due to illness, grade retention, and moving, 26 children (8.02%) were absent at the time of follow-up assessments. Compared to children who stayed in, those who dropped out did not differ in sex (χ^2^ (1, 323) = 0.59, *p* = 0.44). However, dropout children had higher ratings of depressive (*t* = 2.12, *p* = 0.04) and aggressive symptoms (*t* = 2.03, *p* = 0.04) at baseline, compared to children with complete data.

In the fall of kindergarten (after the first assessment), approximately half of the children received a preventive intervention program (Promoting Alternative Thinking Strategies (PATHS), Kusché & Greenberg, 1994). Schools were randomly assigned to the intervention or control condition except for 4 schools who wanted to participate only if they were allowed to express their own preference (two of these schools preferred to be in the intervention condition while the other two schools preferred to be in the control condition). PATHS was designed to improve children’s social-emotional competences by teaching children the skills in identifying and interpreting emotions correctly, including improving social interaction with classmates, and improving social problem solving. It prescribes 29 lessons during kindergarten. Implementation fidelity was assessed by asking teachers in the intervention group to record lessons they had completed. The overall completeness was 40% (range 8% – 59%). Despite the high variation and the overall relatively low rate of program adherence, an intention to treat approach was used when analyzing intervention effects.

### Measures

*Low Social Preference* was assessed with peer nominations. At each time of assessment, children were asked to nominate an unlimited number of classmates whom they liked most and whom the liked least (Coie et al. 1982). Children were interviewed one-on-one by independent interviewers and were instructed to point at photos of classmates for nominations. Peer nomination scores of each child were reported by multiple children, and were obtained and computed completely independent of teacher reports. To normalize the scores between classes with different sizes, the raw nomination scores were divided by the number of children in each class minus 1 (because children were not allowed to nominate themselves; Coie et al. 1982). A low social preference score was generated by subtracting the normalized “liked most” score from the normalized “liked least” score, resulting in a scale ranging from +1 to −1. High scores indicate that children were less liked and more disliked by peers.

*Depressive Symptoms* and *Aggressive Behavior* were rated by teachers using the Problem Behavior at School Interview (PBSI; Erasmus MC, Problem Behavior at School Interview, unpublished manuscript). For each child, the rating at T1 was done by the kindergarten teacher and the ratings at T2 and T3 were done by the first grade elementary school teacher. Assessments at T1 and T3, but not T2, were included in model analyses in the present study, to prevent associations due to shared-method effects (ratings at T2 and T3 share the same rater) and thus increase the validity of the results by using different raters for the repeated assessments of depression and aggression. Depressive and aggressive symptoms were rated on a 5-point scale ranging from *never applicable* to *often applicable*. For depressive symptoms, three items were measured: “Is unhappy or depressed”, “Is indifferent, apathetic and unmotivated”, and “Does not take pleasure in activities”. Cronbach’s alpha for the depressive symptoms scale was 0.73 at T1, and 0.81 at T3. For aggressive behavior, five items were measured: “Threatens other people”, “Starts fights”, “Pushes other children or puts them at risk”, “Bullies, or is mean to others”, and “Attacks others physically”. Cronbach’s alpha for the aggressive behavior scale was 0.92 at T1 and 0.91 at T3. For both variables, high scores indicate high levels of symptoms. Latent constructs were used for both depressive symptoms and aggressive behavior in the structural equation model to account for potential measurement error and improve model fit.

*Change in prosocial behavior* from T1 to T2 was used as a buffering variable in the model analyses of the present study. This variable was calculated based on the level scores of prosocial behavior at T1 and T2. The levels of prosocial behavior at T1 and T2 were measured by the prosocial behavior scale from the Social Experiences Questionnaire-Teacher Report (SEQ-T; Cullerton-Sen and Crick 2005). Similar to the reporting of PBSI, the kindergarten teacher reported at T1 and the first grade elementary school teacher reported at T2. Four items were rated on a 5-point Likert scale from *never applicable* to *often applicable*: “Is nice to other children”, “Helps other children”, “Comforts a child who is crying or sad”, and “Invites other children to play together”. The mean item scores were used, resulting in a scale score ranging from 0 to 4. High scores indicate high levels of prosocial behavior. Cronbach’s alpha was 0.78 at T1 and 0.74 at T2. The change in prosocial behavior of children from T1 to T2 was represented by the unstandardized residual score (URS) obtained by regressing the level score of prosocial behavior at T2 on its level score at T1. The URS not only adjusts for measurement errors (Dalecki and Willits 1991), but also excludes the effect of natural maturation of prosocial behavior on individuals’ behavioral changes over time (Knafo and Plomin 2006). Thus, a score of 0 means that the increase in prosocial behavior exhibited by the child is the same as the average level of change among this sample, and a positive score indicates that the increase in prosocial behavior is above the average trend. A negative score indicates that the change in prosocial behavior is below the average level of change, which can be either decrease or less-than-average increase in level of prosocial behavior.

*Intervention status* and *Sex* were dummy coded. The intervention status was coded as 0 = no intervention/control, 1 = intervention. Sex were coded as 0 = boy, 1 = girl.

### Data Analyses

**Fig. 1:**
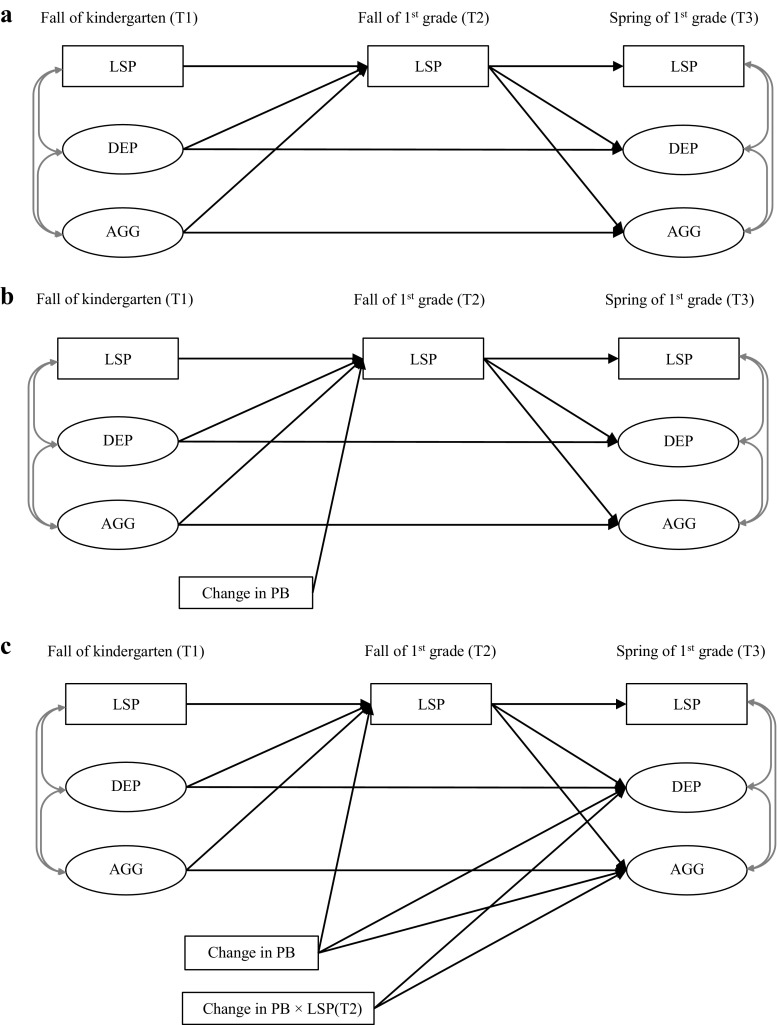
The conceptual model of data analyses in the present study. PB = prosocial behavior. LSP = low social preference. DEP = depressive symptoms. AGG = aggressive behavior

We started by fitting a model estimating the effect of low social preference on development of depressive and aggressive symptoms (see Fig. 1a for the conceptual model). The model contained paths from low social preference at T1 to depressive symptoms and aggressive behavior at T3 via low social preference at T2. In this model, depressive symptoms and aggressive behavior at T1 were included to estimate the effect of low social preference on the changes in depressive and aggressive symptoms from T1 to T3. This model thus estimated the effect of (prolonged) low social preference on the development of depressive and aggressive symptoms, without accounting for the effect of change in prosocial behavior. Correlations between parallel-assessed variables were also included in the model.

Next, we tested our hypotheses by adding the change in prosocial behavior from T1 to T2 into the model. Corresponding to the two hypotheses, two effects of change in prosocial behavior were added and tested one after another. For the first one, the effect of change in prosocial behavior on the development of social preference from T1 to T2, we added a path from change in prosocial behavior from T1 to T2 to low social preference at T2 (see Fig. 1b).

For the second effect, we added change in prosocial behavior from T1 to T2 as a moderator of the prospective links from low social preference at T2 to the development of depressive symptoms and aggressive behavior from T1 to T3. This was done by adding the product of change in prosocial behavior and low social preference at T2 as a predictor of depressive and aggressive symptoms at T3 (see Fig. 1c), as well as the main effect of change in prosocial behavior for controlling purpose (Preacher et al. 2007).

To examine whether our hypothesized effects were different for boys and girls, sex was added as a moderator in both of our hypothesized associations. To test sex effects for the first hypothesis, the product of sex and change in prosocial behavior was regressed on the path from change in prosocial behavior to low social preference at T2. For the second hypothesis, the product of sex, change in prosocial behavior, and low social preference at T2 was regressed on the path from low social preference at T2 to depressive and aggressive symptoms at T3 (after adding all two-way interaction terms; Dawson and Richter 2006). To examine whether our hypothesized effects for children in the intervention condition were different from those in control condition, the effect of intervention status was also estimated, in the same way as the effect of sex was tested.

Model fit was determined based on the comparative fit index (CFI), Tucker-Lewis index (TLI; acceptable value >0.90 for both; Bentler 1990), and the root mean square error of approximation (RMSEA; acceptable values <0.08; Browne and Cudeck 1992). The structural models were fitted in Mplus 7.31 (Muthén & Muthén, 1998–2015). Standard errors of all paths were adjusted for clustering to data using a sandwich estimator (Williams 2000). Maximum likelihood estimation with robust standard errors was adopted in order to account for the non-normal distributions and missing data in the model.

## Results

### Descriptive Statistics

The means and standard deviations of all variables are presented in Table 1. Repeated measures analyses of variance (ANOVAs) indicated that across time boys showed lower levels of social preference (η^2^ = 0.07) and prosocial behavior (η^2^ = 0.03), and higher levels of aggressive behavior (η^2^ = 0.06) than girls. There were no gender differences on depressive symptoms and change in prosocial behavior. Repeated ANOVAs showed no effect of intervention on low social preference, depressive symptoms and aggressive behavior (in Table 1). However, a t-test with independent samples showed that change in prosocial behavior in the intervention condition was higher than in the control condition (Cohen’s *d* = 0.59).

**Table 1:** Means and standard deviations of study variables

		Sex			Intervention		
	Whole sample	Boy	Girl		PATHS	Control	
	*M* (*SD*)	*M* (*SD*)	*M* (*SD*)		*M* (*SD*)	*M* (*SD*)	
*N*	324	176	148	–	158	166	–
1. Low social preference T1	−0.02 (1.55)	0.33 (1.64)	−0.44 (1.33)	*F* = 19.93^***^	−0.03 (1.52)	−0.02 (1.59)	*F* = 0.02
2. Low social preference T2	0.01 (1.72)	0.37 (1.82)	−0.42 (1.48)		−0.03 (1.69)	0.04 (1.74)	
3. Low social preference T3	0.01 (1.69)	0.34 (1.76)	−0.39 (1.51)		−0.02 (1.71)	0.03 (1.67)	
4. Depressive symptoms T1	0.82 (0.59)	0.84 (0.60)	0.80 (0.58)	*F* = 1.49	0.92 (0.60)	0.73 (0.56)	*F* = 1.63
5. Depressive symptoms T3	0.75 (0.64)	0.80 (0.66)	0.69 (0.62)		0.77 (0.65)	0.73 (0.64)	
6. Aggressive behavior T1	0.72 (0.76)	0.93 (0.83)	0.45 (0.58)	*F* = 32.74^***^	0.89 (0.86)	0.55 (0.61)	*F* = 1.61
7. Aggressive behavior T3	0.49 (0.62)	0.63 (0.68)	0.32 (0.48)		0.61 (0.67)	0.38 (0.55)	
8.Change in prosocial behavior	0.00 (0.62)	−0.04 (0.62)	0.05 (0.62)	*t* = 2.00	0.19 (0.59)	−0.16 (0.61)	*t* = 4.91^***^

^*^ *p* < 0.05, ^***^ *p* < 0.001

Bivariate correlations of all studied variables are presented in Table 2. Significant correlations between low social preference scores across time were observed. Low social preference was positively correlated with depressive symptoms and aggressive behavior across time. Change in prosocial behavior from T1 to T2 had a negative correlation with low social preference across time. It also negatively correlated with depressive and aggressive symptoms at T3, but did not correlated with any of them at T1.

**Table 2:** Bivariate correlations between study variables

	1	2	3	4	5	6	7
1. Low social preference T1	–						
2. Low social preference T2	0.51^**^	–					
3. Low social preference T3	0.48^**^	0.65^**^	–				
4. Depressive symptoms T1	0.19^**^	0.21^**^	0.18^**^	–			
5. Depressive symptoms T3	0.20^**^	0.18^**^	0.25^**^	0.17^**^	–		
6. Aggressive behavior T1	0.44^**^	0.37^**^	0.31^**^	0.42^**^	0. 51^**^	–	
7. Aggressive behavior T3	0.41^**^	0.46^**^	0.47^**^	0.22^**^	0.46^**^	0.48^**^	–
8.Change in prosocial behavior	−0.14^**^	−0.21^**^	−0.22^**^	0.01	−0.34^**^	−0.02	−0.28^**^

^**^ *p* < 0.01

### Prosocial Behavior, Social Preference and Depressive and Aggressive Symptoms

The baseline model (Fig. 1a) containing an indirect path from low social preference at T1 to depressive symptoms and aggressive behavior at T3 via low social preference at T2 was fitted first. This model showed an acceptable fit to the data (CFI = 0.94, TLI = 0.93, RMSEA = 0.05). The paths from low social preference at T2 to depressive symptoms (*B* = 0.08, *SE* = 0.03, β = 0.15, *p* = 0.01) and aggressive behavior (*B* = 0.17, *SE* = 0.04, β = 0.34, *p* < 0.001) at T3 were both significant. Also, significant indirect pathways were found from low social preference at T1 to depressive symptoms at T3 (*B* = 0.03, *SE* = 0.01, β = 0.06, *p* = 0.01) and aggressive behavior at T3 (*B* = 0.07, *SE* = 0.02, β = 0.14, *p* < 0.001) via low social preference at T2. The results indicate that children’s low social preference at the beginning of kindergarten predicted their low social preference one year later in the first grade, which consequently predicted increases in symptoms of depression and aggression from kindergarten to the end of first grade elementary school.

**Fig. 2:**
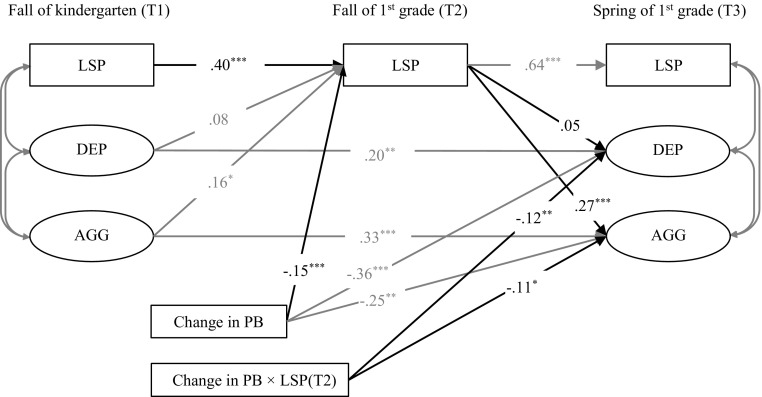
Effect of low social preference on depressive symptoms and aggressive behavior and the effect of change in prosocial behavior. ^*^ *p* < 0.05, ^**^ *p* < 0.01, ^***^ *p* < 0.001. PB = prosocial behavior. LSP = low social preference. DEP = depressive symptoms. AGG = aggressive behavior

To test our hypotheses, we included change in prosocial behavior from T1 to T2 in the model. Corresponding to the two hypotheses, the change in prosocial behavior score was added as a predictor of low social preference at T2, and also as a moderator on the paths from low social preference at T2 to depressive and aggressive symptoms at T3, respectively. With respect to the first hypothesis, results (see Fig. 2) showed a negative and significant effect of change in prosocial behavior on low social preference at T2. This negative link indicates that increases in children’s prosocial behavior predicted reductions in rates of low social preference from T1 to T2. Further analyses showed no significant sex difference (*B* = 0.16, *SE* = 0.10, β = 0.11, *p* = 0.10) or intervention effect (*B* = −0.05, *SE* = 0.10, β = −0.03, *p* = 0.61) on this path.

To test our second hypothesis, change in prosocial behavior from T1 to T2 was added as the moderator of the paths from low social preference at T2 to depressive and aggressive symptoms at T3, respectively. Results indicated that change in prosocial behavior from T1 to T2 significantly modified the link from low social preference at T2 to the development of depressive symptoms as well as of aggressive behavior from T1 to T3. Further analysis showed no significant sex difference in these effects on the link from peer preference to depressive (*B* = −0.05, *SE* = 0.07, β = −0.05, *p* = 0.53) nor aggressive symptoms (*B* = −0.05, *SE* = 0.04, β = −0.05, *p* = 0.24). Also, no significant intervention effect was found in the pathway toward depressive symptoms (*B* = 0.02, *SE* = 0.07, β = 0.02, *p* = 0.80). However, the pathway toward aggression was different for intervention and control group children (*B* = −0.13, *SE* = 0.04, β = −0.16, *p* = 0.001). A breakdown of this effect showed that for control group children, there was no significant modifying effect of change in prosocial behavior (*B* = −0.02, *SE* = 0.02, β = −0.05, *p* = 0.34) in the link between low social preference at T2 and aggressive behavior at T3. However, for children in the intervention group, change in prosocial behavior significantly modified the link from low social preference at T2 to aggressive behavior at T3 (*B* = −0.16, *SE* = 0.04, β = −0.24, *p* < 0.001).

To break down the interaction effect of low social preference at T2 by change in prosocial behavior from T1 to T2, the interaction term was probed by estimating the effects of above-average increase in prosocial behavior (1 *SD* above the mean), average increase (mean) and below-average increase or decrease in prosocial behavior (1 *SD* below the mean), respectively, in the prediction of low social preference at T2 on the development of depressive and aggressive symptoms (Holmbeck 2002). For depressive symptoms, the probing model was fitted with the whole sample. Results showed that low social preference at T2 was positively associated with development of depression for children with low (−1 SD) scores of increase in their prosocial behavior in kindergarten (see Table 3). This link was, however, not significant for children with a high increase (+1 SD) or those who followed the average trend (mean) in their prosocial behavior. Furthermore, results for the indirect pathways showed that the indirect link from low social preference at T1 to T2, and further to depressive symptoms was also significant only for children with a low increase in prosocial behavior (−1 SD) and non-significant for children with an average (mean) or high increase in prosocial behavior (+1 SD) from T1 to T2.

**Table 3:** Effect of low social preference on the development of depressive symptoms (in the total sample) and aggressive behavior (in the intervened group) with different levels of change in prosocial behavior

	Change in Prosocial Behavior								
	Increasing (+1SD)			Average level			Not increasing (-1SD)		
	*B*	*SE*	β	*B*	*SE*	β	*B*	*SE*	β
Direct effects									
LSP (T2) → DEP (T3)	−0.05	0.04	−0.09	0.02	0.03	0.05	0.09	0.03	0.17^**^
LSP (T2) → AGG (T3)	0.07	0.06	0.14	0.21	0.04	0.38^***^	0.35	0.05	0.53^***^
Indirect effects									
LSP (T1) → LSP (T2) → DEP (T3)	−0.02	0.02	−0.04	0.01	0.01	0.02	0.03	0.01	0.07^**^
LSP (T1) → LSP (T2) → AGG (T3)	0.03	0.03	0.05	0.08	0.03	0.14^**^	0.14	0.04	0.20^***^

*LSP*, low social preference; *DEP*, depressive symptoms; *AGG*, aggressive behavior

^**^ *p* < 0.01, ^***^ *p* < 0.001

For aggressive behavior, since the modifying effect was only significant for children in the intervention group, probing models were fitted only within this group. Results showed that the positive association between social preference and aggressive behavior was significant for children who had a low score of increase in their prosocial behavior (−1 SD) and who followed the average trend (mean), but not significant for those who had a high score (+1 SD).

## Discussion

The present study examined the effect of change in prosocial behavior on the link between low social preference and the development of depressive and aggressive symptoms in a community sample of 324 children from kindergarten to the end of first grade elementary school. In line with our hypotheses, we found that poor peer appraisal in kindergarten predicted poor peer appraisal in early elementary school, which in turn predicted increase in symptoms of depression and aggression. We also found that increase in prosocial behavior during kindergarten improved children’s appraisal among peers at the beginning of the first grade. Also, change in prosocial behavior during kindergarten modified the effect of low social preference on the development of depressive and aggressive symptoms from kindergarten to the end of the first grade in elementary school. Children’s low social preference at the beginning of first grade elementary school was not associated with the development of depression and aggression when children’s prosocial behavior increased more than average level (although the effect might be limited to a certain group, to be discussed below). However, when children with a low social position failed to increase their prosocial behavior during kindergarten, their prolonged low social preference predicted increases in depressive symptoms and aggressive behavior from kindergarten to the first grade in elementary school.

Based on our findings, we suggest that the protective effect of increase in prosocial behavior functions in two parts. First, increase in prosocial behavior seems to improve children’s general social position among peers. Our results showed that increase in prosocial behavior during kindergarten were linked to increases in social preference at the beginning of the first grade. This finding indicates that behaving in an extra prosocial manner increased children’s social preference level. This is in line with previous findings showing that prosocial behavior helped in improving children’s interpersonal relationships (Caputi et al. 2012; Crick 1996). In terms of studies within the frame of ostracism theory, our findings also offered new evidence. Previous studies confirmed that under experimental conditions children exhibited more prosocial behaviors when being rejected (Song et al. 2015; Watson-Jones et al. 2016), but these studies did not test the influence of this adjustment. The present study confirmed the effect of prosocial adjustment among children in a real-life social environment. Second, the protective effect of an increase in prosocial behavior appeared to work through mitigation of the link between social preference and the development of both depressive and aggressive symptoms of children because of its positive effect on the child’s social preference. Our findings add new information about the protective effect of prosocial behavior on the development of psychopathologic symptoms in the context of the peer relationship. Studies in the field of child interpersonal relationships in school found that having supportive relations with teachers and friends buffered the negative effect of being rejected or excluded by peers (Laursen et al. 2007; Spilt et al. 2014). This study presented a new angle by investigating the buffering effect from children’s own behaviors.

Moreover, our findings show that increasing prosocial behavior prevents children staying at a low sociometric position among peers, which in turn helps to reduce their risk of developing depressive and aggressive symptoms. Previous studies showed that prolonged poor peer preference predicted depressive and aggressive symptoms (Burks et al. 1995; Burt and Roisman 2010; Ladd and Troop-Gordon 2003; Van Lier and Koot 2010). This study extends this knowledge by confirming these previous findings while showing simultaneously that this pathway depended upon children’s ability to change their behavior in a prosocial manner. Our findings also suggest that the indirect pathways from low peer preference to depression and aggression may be differentially sensitive to change in prosocial behavior. The present study found a significant buffering effect of change in prosocial behavior on the pathways to both depressive symptoms and aggressive behavior. However, while the buffering effect on depressive symptoms was evident for many children, including those with average and high levels of change, probing results of aggression showed that in order to buffer the effect of low social preference on aggression, children need to improve their prosocial behavior at least above the average trend. This indicates a somewhat higher threshold for the buffering effect towards aggression compared to effects towards depression. This suggests that in order to prevent the impact of peer social stresses on psychopathological symptoms, different levels of prosocial adjustment may be required to buffer against different target symptoms.

We also examined whether boys and girls experienced the same positive effect from increase in prosocial behavior in terms of the development of psychopathologic symptoms. Results showed no significant sex differences on either of our hypothesized effects. This suggests that the buffering effect of increase in prosocial behavior is not gender-specific. Despite the sex differences in levels of peer problems and psychopathologic symptoms, no differences were found in the effect from change in prosocial behavior to social preference and the link from social preference to depressive and aggressive symptoms. This is in line with several previous studies that failed to find significant sex differences in the longitudinal associations among these structures (Dodge et al. 2003; Obsuth et al. 2015; Van Lier and Koot 2010). However, these previous studies did not test the modifying effect of change in prosocial behavior in the link between social preference and maladaptation. Moreover, other studies found significant associations between peer problems, prosocial behavior, and internalizing problems only for boys but not for girls (Burks et al. 1995; Griese and Buhs 2013).

The present study found a significant buffering effect of change in prosocial behavior on the pathway to aggression only among children who were in the PATHS intervention condition. We have to be cautious in our interpretation of this effect because the implementation fidelity of the program was fairly low. In fact, it would need to be replicated to draw stronger conclusions on effects of the intervention. However, PATHS might provide a possible explanation as to why change in prosocial behavior prevented social preference to link to aggression among children in the intervention group. The PATHS program aims at teaching children skills to regulate their behavior (Kusché & Greenberg, 1994), so this may have reduced their tendency to develop aggressive behavior more than the tendency to develop depressive symptoms in the context of experiencing social stressors, which explain why the buffering effect of increase in prosocial behavior on the development of aggressive behavior in this group was only significant for those who showed a strong increase.

This study has important theoretical implications. The protective effect of increase in prosocial behavior and its mechanism were proposed in the ostracism theory (Williams 2009). Previous studies within this theoretical framework were mainly conducted with adults and under experimental conditions (e.g., Wesselmann et al. 2012). The present study, via investigating children who just entered kindergarten, lends support to ostracism theory and shows that the theory also holds at a much younger age. On the other hand, findings of the present study indicate that some adjustments may be required when applying ostracism theory to child psychopathology studies. For example, in ostracism theory, mainly depression, and to a lesser extent aggression, were proposed as long-term developmental outcomes of persistent peer problems (Williams 2009). However, in child psychopathologic studies, previous studies showed overlaps of effect from peer problems to the development of both depression and aggression (Burt and Roisman 2010; Ladd 2006; Van Lier and Koot 2010). This is also supported by the present study. Thus, when adapting the theory to the child development area both depression and aggression may need to be considered as outcomes of ostracism.

Findings of the present study also have implications for future researches and preventive interventions. In view of the buffering effect on the links from peer problems to psychopathology, our findings offer support for the importance of improving prosocial behavior among children who are at risk in the peer context. Identifying characteristics of children, or of the children’s environment that make them less capable of changing their prosocial behavior is warranted. Future studies could add relevant information by identifying child characteristics including social cognitive features such as social beliefs (Chen et al. 2012), as well as factors in the school environment, such as sensitivity of teachers (Eisenberg et al. 1981). Findings from our study suggest that preventive intervention programs may focus on teaching children, especially those experiencing rejection, about the skills needed for prosocial behavior (Eisenberg et al. 2015). Our findings also suggest that such interventions should be implemented as early as the kindergarten year to equip children with the skills necessary for coping with social difficulties and improving their peer social position. In addition, findings of our study suggest that (lack of) making needed change in prosocial behavior in response to peer rejection can also be taken as a behavioral marker that helps in identifying children with elevated risk of developing psychopathological symptoms in the context of experiencing peer-related social stressors. Finally, because the implementation fidelity of the intervention in this study was less than optimal, further studies may explore how using PATHS could possibly improve children’s prosocial behavior and affect the pathways examined in this study.

### Limitations and Conclusion

The present study has a number of limitations. First, the majority of the children came from a Dutch/Caucasian ethnic background, which questions whether our findings can be generated to groups with more culturally diverse populations. Prosocial behavior is seen as a personal decision in western culture, while in collectivistic cultures it is usually interpreted as an obligatory choice (Chen 2012; Miller 1994). Cultural differences in the evaluations of prosocial behavior may affect its meaning in social interactions, which may further influence its effects on interpersonal relationships. Also, the schools that participated in the present study were not randomly selected. The percentage of children with minority ethnicity background in our sample (4.8%) is lower than it is in the general population in the Netherlands (>20%, based on data from the organization of Statistics Netherlands). Future studies including children from more diverse ethnic background may be needed to test whether our findings could be generalized to the broader population.

In conclusion, this study, with a longitudinal design in a real social interaction context, found that increase in prosocial behavior as early as in the kindergarten can protect children from developing prolonged low sociometric appraisal among peers, which further protects them from developing depressive and aggressive symptoms. Meanwhile, stronger prosocial adjustments might be required to prevent aggression compared to depression. The findings offer support for the importance of improving prosocial behavior in peer context in terms of a buffering effect on the links from peer problems to psychopathology.

## References

[CR1] Bentler PM (1990). Comparative fit indexes in structural models. Psychological Bulletin.

[CR2] Bilsky SA, Cole DA, Dukewich TL, Martin NC, Sinclair KR, Tran CV (2013). Does supportive parenting mitigate the longitudinal effects of peer victimization on depressive thoughts and symptoms in children?. Journal of Abnormal Psychology.

[CR3] Boivin M, Hymel S, Bukowski WM (1995). The roles of social withdrawal, peer rejection, and victimization by peers in predicting loneliness and depressed mood in childhood. Development and Psychopathology.

[CR4] Bollmer JM, Milich R, Marris MJ, Maras MA (2005). A friend in need: The role of friendship quality as a protective factor in peer victimization and bullying. Journal of Interpersonal Violence.

[CR5] Brendgen M, Vitaro F, Turgeon L, Poulin F (2002). Assessing aggressive and depressed children’s social relations with classmates and friends: A matter of perspective. Journal of Abnormal Child Psychology.

[CR6] Browne MW, Cudeck R (1992). Alternative ways of assessing model fit. Sociological Methods & Research.

[CR7] Burks VS, Dodge KA, Price JM (1995). Models of internalizing outcomes of early rejection. Development and Psychopathology.

[CR8] Burt KB, Roisman GI (2010). Competence and psychopathology: Cascade effects in the NICHD study of early child care and youth development. Development and Psychopathology.

[CR9] Caputi M, Lecce S, Pagnin A, Banerjee R (2012). Longitudinal effects of theory of mind on later peer relations: The role of prosocial behavior. Developmental Psychology.

[CR10] Chen X (2012). Culture, peer interaction, and socioemotional development. Child Development Perspectives.

[CR11] Chen Z, DeWall CN, Poon K-T, Chen E-W (2012). When destiny hurts: Implicit theories of relationships moderate aggressive responses to ostracism. Journal of Experimental Social Psychology.

[CR12] Cicchetti D, Natsuaki MN (2014). Multilevel developmental perspectives toward understanding internalizing psychopathology: Current research and future directions. Development and Psychopathology.

[CR13] Cillessen AHN, Mayeux L (2004). From censure to reinforcement: Developmental changes in the association between aggression and social status. Child Development.

[CR14] Coie JD, Dodge KA, Coppotelli H (1982). Dimensions and types of social status: A cross-age perspective. Developmental Psychology.

[CR15] Crick NR (1996). The role of overt aggression, relational aggression, and prosocial behavior in the prediction of children’s future social adjustment. Child Development.

[CR16] Cullerton-Sen C, Crick NR (2005). Understanding the effects of physical and relational victimization: The utility of multiple perspectives in predicting social-emotional adjustment. School Psychology Review.

[CR17] Dalecki M, Willits FK (1991). Examining change using regression analysis: Three approaches compared. Sociological Spectrum.

[CR18] Dawson JF, Richter AW (2006). Probing three-way interactions in moderated multiple regression: Development and application of a slope difference test. Journal of Applied Psychology.

[CR19] DeRosier MEK, Janis B (1994). Children’s academic and behavioral adjustment as a function of the chronicity and proximity of peer rejection. Child Development.

[CR20] Dodge KA, Lansford JE, Burks VS, Bates JE, Pettit GS, Fontaine R, Price JM (2003). Peer rejection and social information-processing factors in the development of aggressive behavior problems in children. Child Development.

[CR21] Eisenberg N, Cameron E, Tryon K, Dodez R (1981). Socialization of prosocial behavior in the preschool classroom. Developmental Psychology.

[CR22] Eisenberg N, Eggum-Wilkens ND, Spinrad TL, Schroeder DA, Graziano WG (2015). The development of prosocial behavior. The Oxford handbook of prosocial behavior.

[CR23] Fontaine RG, Yang C, Burks VS, Dodge KA, Price JM, Pettit GS, Bates JE (2009). Loneliness as a partial mediator of the relation between low social preference in childhood and anxious/depressed symptoms in adolescence. Development and Psychopathology.

[CR24] Gooren EMJC, van Lier PAC, Stegge H, Terwogt MM, Koot HM (2011). The development of conduct problems and depressive symptoms in early elementary school children: The role of peer rejection. Journal of Clinical Child & Adolescent Psychology.

[CR25] Griese ER, Buhs ES (2013). Prosocial behavior as a protective factor for children’s peer victimization. Journal of Youth and Adolescence.

[CR26] Haselager GJT, Cillessen AHN, Van Lieshout CFM, Riksen-Walraven JMA, Hartup WW (2002). Heterogeneity among peer-rejected boys across middle childhood: Developmental pathways of social behavior. Developmental Psychology.

[CR27] Hoglund WLG, Chisholm CA (2014). Reciprocating risks of peer problems and aggression for children’s internalizing problems. Developmental Psychology.

[CR28] Holmbeck GN (2002). Post-hoc probing of significant moderational and mediational effects in studies of pediatric populations. Journal of Pediatric Psychology.

[CR29] Jiang, X. L., & Cillessen, A. H. N. (2005). Stability of continuous measures of sociometric status: A meta-analysis. *Developmental Review, 25, 25*(1). 10.1016/j.dr.2004.08.008.

[CR30] Keiley MK, Bates JE, Dodge KA, Pettit GS (2000). A cross-domain growth analysis: Externalizing and internalizing behaviors during 8 years of childhood. Journal of Abnormal Child Psychology.

[CR31] Knafo A, Plomin R (2006). Prosocial behavior from early to middle childhood: Genetic and environmental influences on stability and change. Developmental Psychology.

[CR32] Kusché, C. A., & Greenberg, M. T. (1994). *The PATHS curriculum*. South Deerfield, MA: Channing-Bete Co.

[CR33] Ladd GW (2006). Peer rejection, aggressive or withdrawn behavior, and psychological maladjustment from ages 5 to 12: An examination of four predictive models. Child Development.

[CR34] Ladd GW, Troop-Gordon W (2003). The role of chronic peer difficulties in the development of children’s psychological adjustment problems. Child Development.

[CR35] Ladd GW, Herald SL, Kochel KP (2006). School readiness: Are there social prerequisites?. Early Education and Development.

[CR36] Lam CB, McHale SM, Crouter AC (2014). Time with peers from middle childhood to late adolescence: Developmental course and adjustment correlates. Child Development.

[CR37] Laursen B, Bukowski WM, Aunola K, Nurmi J-E (2007). Friendship moderates prospective associations between social isolation and adjustment problems in young children. Child Development.

[CR38] Miller JG (1994). Cultural diversity in the morality of caring: Individually oriented versus duty-based interpersonal moral codes. Cross-Cultural Research.

[CR39] Modin B, Östberg V, Almquist Y (2010). Childhood peer status and adult susceptibility to anxiety and depression: A 30-year hospital follow-up. Journal of Abnormal Child Psychology.

[CR40] Muthén L. K, & Muthén, B. O. (1998–2015). *Mplus user’s guide* (7th ed.). Los Angeles, CA: Muthén & Muthén.

[CR41] Obsuth, I., Eisner, M. P., Malti, T., & Ribeaud, D. (2015). The developmental relation between aggressive behaviour and prosocial behaviour: A 5-year longitudinal study. *BMC Psychology, 3*. 10.1186/s40359-015-0073-4. 10.1186/s40359-015-0073-4 PMC4440499 26000166

[CR42] Parker JG, Rubin KH, Erath SA, Wojslawowicz JC, Buskirk AA, Cicchetti D, Cohen DJ (2006). Peer relationships, child development, and adjustment: A developmental psychopathology perspective. Developmental psychopathology.

[CR43] Preacher KJ, Rucker DD, Hayes AF (2007). Addressing moderated mediation hypotheses: Theory, methods, and prescriptions. Multivariate Behavioral Research.

[CR44] Rubin KH, Bukowski WM, Parker JG, Damon W, Lerner RM (2006). Peer interactions, relationships, and groups. Handbook of child psychology.

[CR45] Song R, Over H, Carpenter M (2015). Children draw more affiliative pictures following priming with third-party ostracism. Developmental Psychology.

[CR46] Spilt JL, van Lier PAC, Leflot G, Onghena P, Colpin H (2014). Children’s social self-concept and internalizing problems: The influence of peers and teachers. Child Development.

[CR47] Sturaro C, van Lier PAC, Cuijpers P, Koot HM (2011). The role of peer relationships in the development of early school-age externalizing problems. Child Development.

[CR48] Van Lier PAC, Koot HM (2010). Developmental cascades of peer relations and symptoms of externalizing and internalizing problems from kindergarten to fourth-grade elementary school. Development and Psychopathology.

[CR49] Watson-Jones RE, Whitehouse H, Legare CH (2016). In-group ostracism increases high-fidelity imitation in early childhood. Psychological Science.

[CR50] Wesselmann ED, Wirth JH, Mroczek DK, Williams KD (2012). Dial a feeling: Detecting moderation of affect decline during ostracism. Personality and Individual Differences.

[CR51] Will G-J, Van Lier PAC, Crone EA, Güroğlu B (2016). Chronic childhood peer rejection is associated with heightened neural responses to social exclusion during adolescence. Journal of Abnormal Child Psychology.

[CR52] Williams RL (2000). A note on robust variance estimation for cluster-correlated data. Biometrics.

[CR53] Williams KD (2007). Ostracism. Annual Review of Psychology.

[CR54] Williams KD (2009). Ostracism: A temporal need-threat model. In M. Zanna. Advances in experimental social psychology.

